# Altered states phenomena induced by visual flicker light stimulation

**DOI:** 10.1371/journal.pone.0253779

**Published:** 2021-07-01

**Authors:** Marie Therese Bartossek, Johanna Kemmerer, Timo Torsten Schmidt

**Affiliations:** 1 Department of Education and Psychology, Freie Universität Berlin, Berlin, Germany; 2 Department of Psychiatry, Psychotherapy and Psychosomatic Medicine, Vivantes Hospital Am Urban und Vivantes Hospital im Friedrichshain, Charité–Universitätsmedizin Berlin, Berlin, Germany; Victoria University of Wellington, NEW ZEALAND

## Abstract

Flicker light stimulation can induce short-term alterations in consciousness including hallucinatory color perception and geometric patterns. In the study at hand, the subjective experiences during 3 Hz and 10 Hz stroboscopic light stimulation of the closed eyes were assessed. In a within-subjects design (N = 24), we applied the Positive and Negative Affect Schedule (mood state), time perception ratings, the Altered State of Consciousness Rating Scale, and the Phenomenology of Consciousness Inventory. Furthermore, we tested for effects of personality traits (NEO Five-Factor Inventory-2 and Tellegen Absorption Scale) on subjective experiences. Such systematic quantification improves replicability, facilitates comparisons between pharmacological and non-pharmacological techniques to induce altered states of consciousness, and is the prerequisite to study their underlying neuronal mechanisms. The resulting data showed that flicker light stimulation-induced states were characterized by vivid visual hallucinations of simple types, with effects strongest in the 10 Hz condition. Additionally, participants’ personality trait of Absorption scores highly correlated with the experienced alterations in consciousness. Our data demonstrate that flicker light stimulation is capable of inducing visual effects with an intensity rated to be similar in strength to effects induced by psychedelic substances and thereby support the investigation of potentially shared underlying neuronal mechanisms.

## Introduction

The experimental investigation of the neuronal mechanisms underlying phenomena like hallucinations and perceptual distortions is of special interest for neuroscientific research. While typically occurring in a pathological context, such phenomena can also be temporarily induced in healthy individuals. If experimentally induced phenomena are similar in their subjective experience to pathologic symptoms, they might also share common neuronal mechanisms. As such, the experimental induction of e.g., hallucinatory experiences and a systematic quantification of accompanying neuronal changes can crucially contribute to a better understanding of neuronal mechanisms involved in diverse psychopathologies.

For experimental research, various methods are available to induce so-called altered states of consciousness (ASCs). ASCs can range from ‘flow states’, ‘dissociative states’, to ‘psychedelic states’ and can include phenomena such as ‘out of body experiences’, ‘ego dissolution’, or ‘hallucinations’ [1]. According to Tart [2], a state can be called an ASC if it diverges in quality and quantity from the individual’s ordinary state of consciousness to an extent that it is no longer perceived as a usual fluctuation. ASCs can be induced by the application of pharmacological methods (e.g., LSD, psilocybin, ketamine) or non-pharmacological methods (e.g., hypnosis, sensory overload, perceptual deprivation) [3]. In the last years, multiple methods to induce ASCs have been utilized in basic human neuroscientific research [1, 4]. In addition, multiple studies and meta-analyses have contributed to a systematic comparison of the phenomena evoked by different induction methods of ASCs [4–6]. One method that has only been marginally recognized by modern neuroscience is the use of flicker light stimulation (FLS). The research discussed in this paper therefore aims to complement a systematic quantification of FLS-induced phenomena to make them comparable to the phenomenology of ASCs induced by other pharmacological and non-pharmacological methods. It thereby encourages future neuroscientific research into the neuronal mechanisms complicit in FLS-induced subjective experiences.

FLS is a non-pharmacological method to induce short-term alterations in consciousness via closed-eye ocular stimulation with stroboscopic, flickering light. FLS is especially associated with hallucinatory perceptions of colors and geometric patterns [7, 8]. Flicker frequencies between 5 Hz and 30 Hz have been reported to evoke flicker-induced visual hallucinations (FIVHs), the content of which participants are not able to volitionally control [9]. Frequencies in the EEG alpha frequency range (around 10 Hz) are reported to have the highest probability to evoke FIVHs [8, 10, 11].

FLS is also known to cause intense and diverse subjective experiences, as well as an increase in neural signal diversity [8]. Regarding the phenomenology of subjective experiences, Ludwig [12] named a distorted time sense among the characteristics of most ASCs, predominantly stating that time is perceived as passing by slower. Further, FLS has been reported to alter perceived sleepiness, alertness, and effort [13]. Positive effects on mood and divergent thinking are depicted in anecdotal reports about FLS in recreational settings.

Several environmental as well as personality factors are thought to be predictive of the extent to which ASCs are experienced. Such potential predictors have been explored for diverse ASCs-inducing methods, including pharmacological ones. Results are not conclusive and predictors of FLS responses are yet unknown. In previous studies, the role of participants’ personality traits for their responses to multimodal Ganzfeld exposure was tested. Multimodal Ganzfeld refers to the homogenization of visual as well as auditory stimulation [14] and has been shown to induce visual pseudo-hallucinatory phenomena like e.g., the perception of colors [15]. One study reported that ASC experiences correlated negatively with the personality factor Conscientiousness [14, 16], while another study did not find such a correlation [15]. As FIVHs share features with descriptions of visual effects induced by Ganzfeld exposure, the potential effects of Conscientiousness on subjective experiences of FLS are of interest. With respect to the pharmacological induction of ASCs, a positive correlation has been found between participants’ trait of Absorption and psilocybin-induced ASC experiences [17]. Absorption is known to correlate with a sub-facet of the personality factor Openness to Experience associated with imaginative involvement [18]. The trait of Absorption also correlates with hypnotizability and perceived hypnotic depth [19]. It can be speculated that Absorption facilitates the entrance into deep ASC experiences [20] and therefore might correlate with FLS-induced effects.

To enable the study of relations between consciousness state alterations and underlying neural mechanisms, a detailed characterization of the phenomenology of subjective experiences during FLS-induced ASCs is required. Such a systematic quantification enables comparisons with the subjective effects evoked by other ASCs induction methods and enhances the understanding of the mechanisms underlying human consciousness (for further information see the Altered States Database [4]). Furthermore, the possibility to draw parallels between methods might help to identify predictors of therapeutically relevant beneficial effects of pharmacologically induced ASCs based on the experimental investigation of non-pharmacological ASCs induction methods [1].

Though many ASCs induction methods have been investigated in a structured manner, there have been few studies focusing on ASCs evoked by FLS [8]. Hence, the main aim of the present study was to structurally assess the phenomenology of subjectively perceived experiences during FLS-induced ASCs. This was achieved with the help of established tools and questionnaires that enable a comparison with other induction methods [1, 4].

With the study at hand, we provide an overview of the subjective experiences induced by FLS. Apart from that, we were interested in comparing the phenomenology of two conditions, using frequencies of 3 Hz and 10 Hz as previously applied by Schwartzman et al. [8]. We presumed the latter to evoke stronger alterations in consciousness and more FIVHs. Further, we hypothesized that participants would experience FLS-evoked distortions of time perception, alterations of mood, and an increase in divergent thinking, with stronger effects in the 10 Hz condition. Additionally, we aimed to test the hypotheses that the personality trait Conscientiousness would negatively, and the personality trait of Absorption would positively correlate with the extent of FLS-induced alterations in consciousness.

## Materials and methods

### Sample

Twenty-four voluntary German native speakers (mean age: 24.0 ± SD 6.4; 19 female, 5 male) without a history of neurological or psychiatric disorders and normal or corrected-to-normal vision participated in the study. As assessed with the Edinburgh Handedness Inventory [EHI] [21], 17 participants were classified as right-, five as mixed- and two as left-handed (*mean laterality quotient* = 54.38 ± *SD* 39.49). Social media and student mailing lists were used for recruitment. Participants were informed about the study aims and background as well as possible risks of FLS before providing written consent. One participant dropped out of the study due to self-reported migraine symptoms after the first experimental session with FLS of 10 Hz. To compensate for the dropout, data from a new participant was acquired. All materials and procedures were in accordance with the Human Participants Guidelines of the Declaration of Helsinki and were approved by the ethics committee of Charité Berlin.

### Study design and procedure

**Fig 1:**
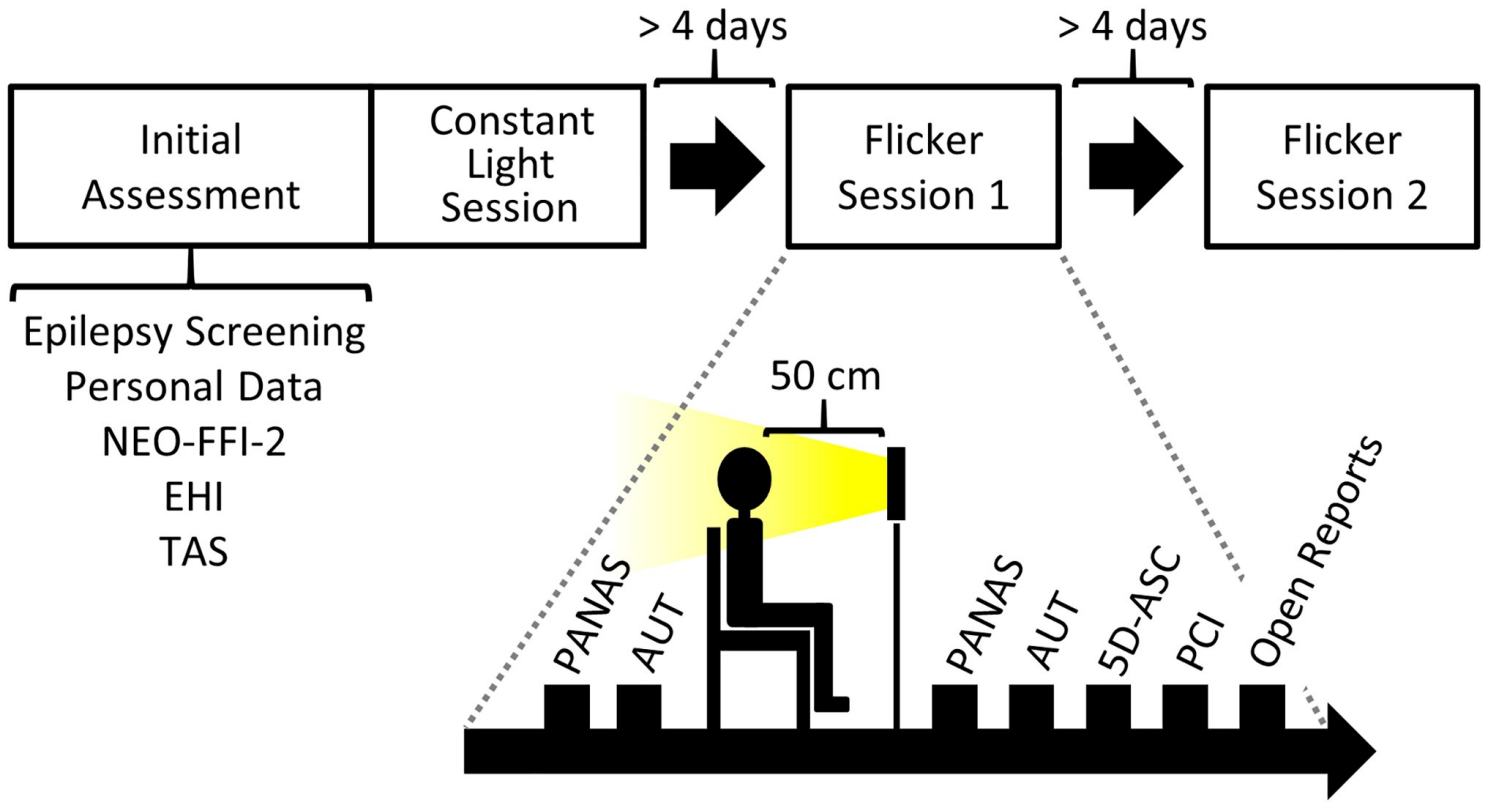
Study design and experimental procedure. The initial assessment included an EEG-based screening for photosensitive epilepsy as well as a psychometric assessment with a personal data inventory, the NEO-FFI-2 personality trait questionnaire [22], the Edinburgh Handedness Inventory [21], and the Tellegen Absorption Scale [19]. The first experimental session [Constant Light Session], in which participants were exposed to constant light for 20 min was conducted immediately after the initial assessment. The subsequent two experimental sessions involved 20 min of exposure to flicker light with frequencies of either 3 Hz or 10 Hz (randomized order). During the light exposure, participants were asked to estimate the time elapsed since the beginning of the exposure either after 12, 14, or 16 min (randomized order over all three sessions within every participant). All sessions consisted of an assessment of mood changes (pre to post) with the Positive and Negative Affect Schedule [23] and divergent thinking with the Alternative Uses Task [24]. Additionally, participants retrospectively rated the subjective effects of the light exposure on the Altered States of Consciousness (ASC) Rating Scale [5D-ASC] [25] and the Phenomenology of Consciousness Inventory [26] and were also asked to describe their experiences in open reports. Experimental sessions [Flicker Session 1 and 2] were conducted with at least four days in between.

To minimize the risk of any aversive effects of FLS, all participants underwent a semi-structured video interview with a psychologist previous to the experiment to ensure that they were not affected by an acute mental or neurological disorder, were not currently or regularly taking any psychotropic medication or drugs and were not pregnant (an English and a German version of the semi-structured interview is provided in S1 Interview). After an initial assessment, including a screening for indications of photosensitive epilepsy based on electroencephalography (EEG), participants partook in an initial light exposure session with constant light to familiarize them with the procedure, questionnaires, and experimental setup. This session was implemented to reduce potential order effects for the two experimental sessions due to a previous study indicating corresponding order effects [15]. On two consecutive sessions (separated by at least 4 days and a maximum of 21 days) participants underwent the same procedures with the two experimental conditions of 3 Hz and 10 Hz visual flicker stimulation which were balanced in order across participants (see Fig 1). Thus, every participant took part in all three sessions, resulting in a within-subjects design. For each participant, all three sessions were conducted at approximately the same time of the day (± 1.5 hours) to ensure that testing conditions would be as constant as possible and to reduce interfering effects of fatigue. All sessions were conducted at the Center for Cognitive Neuroscience Berlin at the Free University of Berlin.

#### Initial assessment and epilepsy screening

After being informed of the study aims, background, and risks, participant characteristics were assessed (Initial Assessment, see Methods). The epilepsy screening, which took place afterward, was based on clinical routines and comprised an EEG recording in resting state (2 min) and measurement during a sequence of FLS episodes of four seconds with frequencies of 1, 3, 10, 20, 30, and 40 Hz. Each flicker frequency episode was presented twice consecutively with breaks of 10 seconds in between. In addition, EEG was recorded during an approximately 2 min period of hyperventilation. The resulting data were screened by a trained expert. Epilepsy screening and questionnaire assessment took approximately 1.25h and was followed by the familiarization session with constant light, following the protocol of the experimental sessions.

#### Sessions

Before light exposure, participants completed the Assessment of Changes set of questionnaires (see Methods). Thereafter, participants were instructed that at one point during the light exposure, they would be tapped on the shoulder and should vocalize their subjective estimation of how much time had passed since the beginning of the exposure. Participants were seated in a reclined armchair facing the stroboscopic light device, which was positioned at an approximate distance of 50 cm. Thereafter, participants were instructed to close their eyes, all lights in the room were turned off, and light exposure was started. Participants wore earplugs throughout the exposure to prevent any auditory distractions. After either 12, 14, or 16 min (randomized order over all three sessions within every participant) of a total duration of 20 min, participants were asked to estimate how much time had elapsed since the beginning of light exposure and their response was recorded. After completion of the light exposure, the Assessment of Changes set was applied again. Subsequently, to assess the phenomenology of participants’ subjective experience during light exposure, participants completed the Post Flicker Assessment set. Each experimental session lasted approximately 1.25 hours in total.

### Materials

#### Flicker light stimulation

For the presentation of FLS, we used the Lucia N°03 stroboscope (Light Attendance GmbH, Innsbruck, Austria). It has been developed to evoke hypnagogic visual impressions by intermittent light stimulation and is used in various recreational contexts. It is equipped with one halogen lamp that is used for constant light stimulation and eight LEDs to apply FLS with high precision timing and luminance via a programmable interface. FLS was applied via the eight LEDs of the stroboscopic light device, each at 700 lm (light color 6300 K). Three light stimulation conditions with a duration of 20 min each were applied in the present study: (1) Constant light stimulation at full intensity halogen light, (2) 3 Hz, and (3) 10 Hz flicker stimulation with 50% ON/OFF times of LED light, as applied by Schwartzman et al. [8].

#### Initial assessment

Participants filled in the Edinburgh Handedness Inventory [21] and the NEO-FFI-2 personality trait questionnaire [22]. Additionally, participants completed a set of items assessing demographic variables and previous experiences with psychedelic drugs and substances as well as certain relaxation techniques including FLS. Finally, the Tellegen Absorption Scale [19] was applied to assess participants’ trait Absorption. This inventory contains 34 items regarding imaginative involvement, each assessed on a five-point Likert scale. Scores can range between 0 and 136.

#### Assessment of changes

To measure changes in the mood state and divergent thinking, we applied the Assessment of Changes set before and after every light exposure:

*Positive and Negative Affect Schedule (PANAS)*: The PANAS [23] assesses current Positive Affect as associated with an active, enthusiastic state of mood on the one hand, and Negative Affect marked by subjectively perceived distress and aversion on the other hand. These two mood dimensions are mainly uncorrelated and are treated as orthogonal factors. Therefore, each factor is assessed with ten items on a five-point Likert scale. In this study, we used a German adaption of the inventory [27].
*Alternative Uses Task (AUT)*: The AUT [24] was applied as an indicator of divergent thinking. In this task, participants are asked to write down as many unusual options to use a certain everyday object (e.g., a spoon) as possible within three min. In total, the task was applied in twelve different versions, each containing a different object. The order was randomized for every participant. The selection of objects was based on former studies using the AUT [28–33]. The results of this test are evaluated by the factors Fluency, Flexibility, and Originality [34]. Two versions of the AUT were applied before and after every light exposure, respectively.

#### Post flicker assessment

To quantify the phenomenology of the FLS-induced state, participants retrospectively answered the following questionnaires after each of the two sessions:

*Altered States of Consciousness (ASC) Rating Scale*: The ASC Rating Scale [5D-ASC] [25] consists of 94 items that assess different characteristics of altered states of consciousness that are hypothesized to be invariant across both pharmacologically (e.g., psilocybin, ketamine) and non-pharmacologically induced ASCs (e.g., sensory deprivation, hypnosis) [25]. Scores on the visual analog scales can range from 0 to 100 percent. The results of the 5D-ASC can be distinguished into five primary dimensions [25]: Oceanic Boundlessness as a dimension indicates positive and enjoyable aspects of the ASC as including the experience of boundary dissolution between oneself and the surroundings as well as dissolution of time and space. It further reflects mystical-type experiences. The dimension Dread of Ego Dissolution is indicative of negative ASC experiences associated with depersonalization and dissociation. Items clustered in the dimension Visionary Restructuralization assess perceptual and imaginational alterations including visual hallucinatory phenomena. Auditory Alterations further reflect changes regarding auditory perceptions and acoustic hallucinations. The fifth dimension Vigilance Reduction refers to the experience of clouded consciousness, sleepiness, or drowsiness.
  The same questionnaire can also be analyzed according to an 11 factors scheme. The resulting factors comprise subscales of the three 5D-ASC dimensions Oceanic Boundlessness, Dread of Ego Dissolution, and Visionary Restructuralization [35]. As FLS is mainly associated with visual hallucinatory perceptions, the subscales Elementary Imagery, indicating the experience of basic visual impressions like colors or patterns (e.g., “I saw regular patterns with closed eyes or in complete darkness”), and Complex Imagery, being indicative of the perception of concrete visual scenes or images (e.g., “I saw whole scenes roll by with closed eyes or in complete darkness”) are of particular interest for the present study. Correspondingly, we report data according to both analysis schemes. The 5D-ASC/11-ASC ranks among the most established questionnaires quantifying ASC experiences and therefore facilitates a comparison of FLS with other ASCs induction methods [4].
*Phenomenology of Consciousness Inventory (PCI)*: The PCI [26] is the advanced version of the Phenomenology of Consciousness Questionnaire [36] and a first version of the PCI [37]. We applied a German version of this retrospective questionnaire [38] that includes 53 items. Different aspects of the subjectively experienced state are assessed on a seven-point Likert scale whose anchor points are characterized by opposed statements. The items are merged into twelve major dimensions, five of which are further divided into 14 minor dimensions. The PCI also enables comparability with other studies investigating the induction of ASCs [1, 4].
*Open Reports*: Aside from standardized inventories, participants were enabled to give a free report of their impressions during FLS.
*Time Awareness and Perceived Speed of Time*: Furthermore, participants were asked to retrogradely rate how frequently they had thought about time (Time Awareness) and how fast time had passed (Perceived Speed of Time) during the exposure on visual analog scales whose anchor points were characterized by opposing statements (Time Awareness: *not at all–extremely often*; Perceived Speed of Time: *extremely slowly*–*extremely fast*). These items have been used in multiple previous studies to assess alterations in time perception [39, 40].
  The instructions for the questionnaires referred to participants’ general experience during light exposure, no specific instructions were given about how to handle potential variance in experiences across time.

### Statistical data analysis

To test if data are normally distributed, we used the Shapiro-Wilk normality test [41]. As this was not the case for the PCI and 5D-ASC questionnaire data and due to the sample being too small to be robust against this violation [42], we chose to perform Wilcoxon signed-rank tests [43] to assess differences between conditions.

As suggested by Dittrich et al. [25], a global score for Altered State of Consciousness (G-ASC) was calculated for every participant. The average score of three dimensions of the 5D-ASC (Oceanic Boundlessness, Dread of Ego Dissolution, Visionary Restructuralization) reflects the etiology-independent core of ASCs and was therefore chosen for calculating correlations with personality traits.

To assess whether flicker frequencies affected the subjectively perceived speed of time, we calculated the ratio θ (θ = estimated/actual duration) as a characteristic value indicative of the extent to which the estimation of the participant deviated from the actual elapsed time. Hence, a ratio of θ < 1 indicates an underestimation and respectively θ > 1 an overestimation of time. This method follows Block et al. [44], as well as Wittmann et al. [45].

The PANAS item scores [23] were assessed pre and post exposure and were separated into two categories of affect valence (Positive Affect and Negative Affect). We conducted 2x2 repeated measures analyses of variance (ANOVA) to test for effects of the point of assessment and experimental condition (3 Hz versus 10 Hz).

The results of the AUT [24] were analyzed in a qualitative manner, following the procedure of Ottemiller et al. [31] as well as the recommendations of Silvia et al. [32]: By use of the software MAXQDA [46], two raters categorized each answer into one of 48 generated categories regarding the alternative use of the item. Inter-rater reliability as assessed with Cohen’s Kappa was very good (κ = .83). Thereafter, each participant’s responses were evaluated concerning the number of responses (Fluency), the number of different categories (Flexibility), and the rareness of these categories (Originality) for each item. To also examine the effects of assessment points and conditions on divergent thinking, we conducted 2x2 repeated measures ANOVAs regarding data of the AUT as well.

Additionally, Pearson product-moment correlations [47] were calculated to characterize the relations between certain assessed variables. Correlations were calculated separately for each experimental condition.

Due to the lack of comparable datasets and the resulting explorative character of the present study, we expected high variance in our data and thus did not exclude outliers from statistical analyses. However, because one participant scored below the tolerated minimum of response consistency within the PCI in one session, as was indicated by a reliability index (h) above two points [26], only N = 23 participants were taken into account in the statistical analysis of this inventory.

Data analyses were performed with R [version 3.6.0] [48], using the packages ez [49], car [50], exactRankTests [51], graphics [48], and stats [48]. Further, version 1.0.143 of the enhancement program RStudio [52] was used.

Participants’ retrospective open reports about the light exposures were examined with qualitative methods: All reports were screened, and categories were formed based on the freely reported subjective experiences. Subsequently, each participant’s reported experiences were classified into the extracted categories separately for each session. It is important to note that each participant’s response could be classified into several categories.

Because constant light sessions were always performed as the first sessions and not included in the randomization of order, we did not use their questionnaire data for comparisons with the experimental flicker conditions, as these data might be biased. However, as we did not expect a bias in time estimation data, we used them for comparisons with those of the experimental 3 and 10 Hz flicker conditions.

## Results

Detailed study data are provided in S1 Data.

### Sample characteristics

There was no bias in personality, as assessed with the NEO-FFI-2 (Neuroticism: 19.17 ± 6.82 (*M* ± *SD*); Extraversion: 30.46 ± 5.67; Openness to Experience: 31.42 ± 5.90; Agreeableness: 37.63 ± 4.78; Conscientiousness: 34.13 ± 8.00). Regarding the TAS [19], participants displayed a mean trait of Absorption score of *M* = 54.5 ± *SD* 26.83 (*range* 5–125; scores can range between 0 and 136). Regarding previous experiences with substance and psychedelic drug consumption, all participants (*n* = 24) reported previous experiences with alcohol, *n* = 16 with cannabis, *n* = 12 with nicotine, *n* = 3 with entactogens, *n* = 2 with opioids, *n* = 2 with stimulants, *n* = 2 with cocaine, *n* = 2 with hallucinogens, *n* = 2 with dissociatives, *n* = 2 with sedatives, and *n* = 0 with dissolvents. None of the participants reported any previous experience with FLS.

### Acute subjective effects of flicker light stimulation

**Fig 2:**
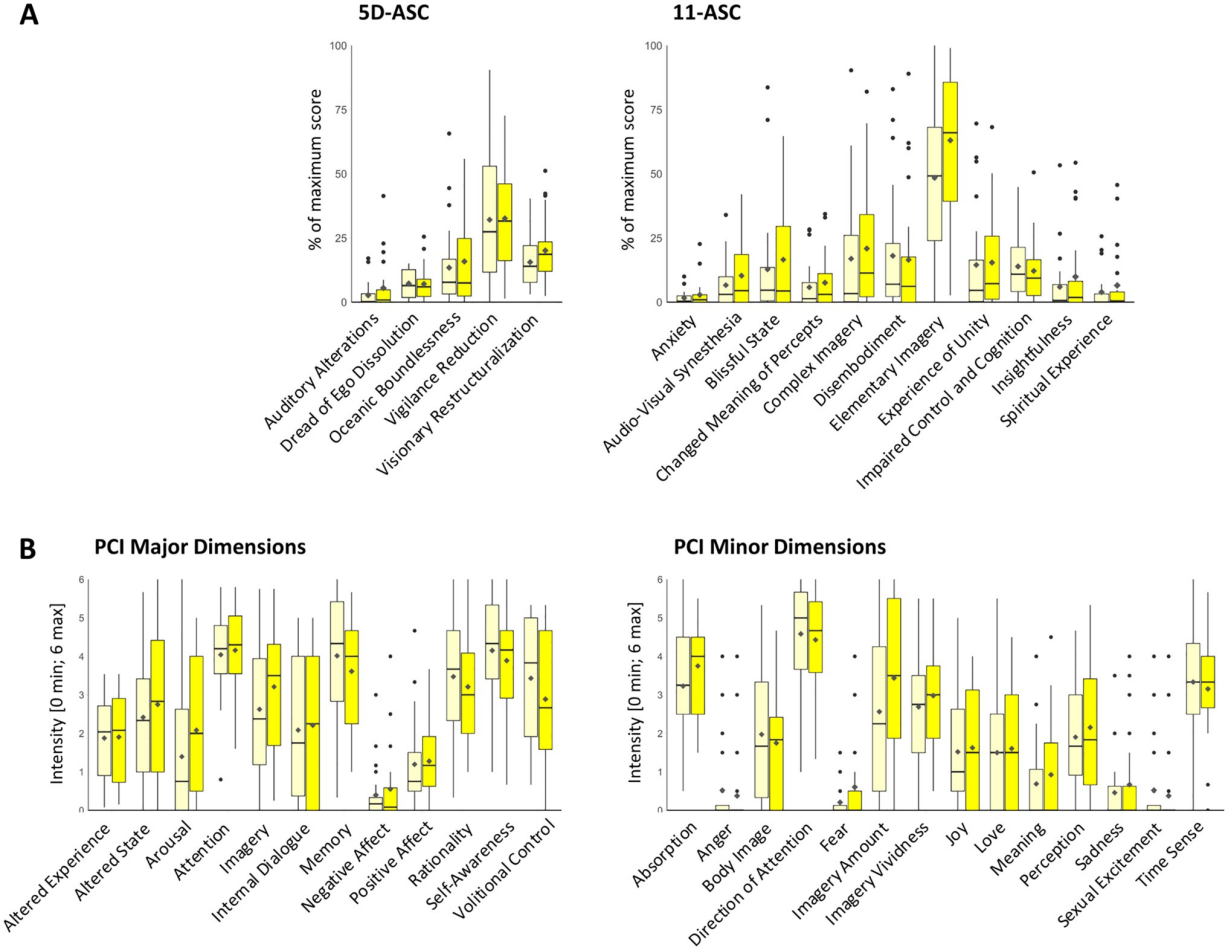
Psychometric assessment of Flicker Light Stimulation (FLS) effects. (A) Retrospective ratings of FLS effects with the Altered States of Consciousness (ASC) Rating Scale [25] analyzed according to the five-dimensional ASC Rating Scale (5D-ASC) scheme and to the 11 subscales of the ASC Rating Scale (11-ASC). (B) Retrospective ratings of FLS effects on the major and minor dimensions of the Phenomenology of Consciousness Inventory [26]. Colors correspond to the 3 Hz (light yellow) and 10 Hz (bright yellow) flicker frequency conditions. Boxplots represent the first to third quartiles as boxes; the horizontal lines within the boxes visualize the median, the yellow points indicate the mean; bars represent 1.5 times the interquartile range of the data with points representing scores outside of that range. None of the differences between the dimension scores of 3 Hz and 10 Hz conditions were significant after Bonferroni-correction of the α-threshold for multiple comparisons.

Results of the 5D-ASC/11-ASC [25] with corresponding means, standard deviations, and p-values for the comparison between the 3 Hz and 10 Hz conditions are provided in Fig 2 and Table 1. We found a descriptive trend for the G-ASC score of the 10 Hz condition (14.2 ± 10.27) being higher than for the 3 Hz condition (12.04 ± 9.66), which however did not exceed significance after Bonferroni correction for multiple testing (*p* = .034). Likewise, the difference between the 3 Hz condition (15.60 ± 10.44) and the 10 Hz condition (20.12 ± 13.13) regarding the dimension of Visionary Restructuralization remained an insignificant trend (*p* = .039).

**Table 1:** Ratings on the dimension of the Altered States of Consciousness (ASC) rating scale [25] within the 3 Hz and 10 Hz conditions.

5D-ASC Dimension	3 Hz		10 Hz		*p*
	*M*	*SD*	*M*	*SD*	
Altered State of Consciousness (global)	12.04	9.66	14.20	10.27	0.034
Auditory Alterations	2.67	4.73	5.48	10.03	0.375
Dread of Ego Dissolution	7.25	5.35	7.09	6.71	0.663
Oceanic Boundlessness	13.41	16.14	15.85	15.85	0.065
Vigilance Reduction	32.13	25.17	32.63	20.12	0.855
Visionary Restructuralization	15.60	10.44	20.12	13.13	0.039
11-ASC Dimension	3 Hz		10 Hz		*p*
	*M*	*SD*	*M*	*SD*	
Anxiety	1.64	2.51	2.81	5.31	0.671
Audio-Visual Synesthesia	6.61	9.11	10.31	12.86	0.117
Blissful State	12.78	21.40	16.56	20.79	0.522
Changed Meaning of Percepts	5.78	9.20	7.56	10.53	0.117
Complex Imagery	16.93	24.61	20.90	26.36	0.169
Disembodiment	18.04	24.70	16.46	24.12	0.961
Elementary Imagery	48.44	27.59	63.05	27.90	0.060
Experience of Unity	14.50	20.51	15.45	18.89	0.756
Impaired Control and Cognition	13.91	12.57	12.14	12.19	0.262
Insightfulness	5.94	12.05	9.89	16.80	0.316
Spiritual Experience	3.87	7.25	6.54	12.66	0.135

*Note*. Differences between conditions were assessed by Wilcoxon signed-rank tests. Data were analyzed according to the five-dimensional ASC Rating Scale (5D-ASC) scheme and to the 11 subscales of the ASC Rating Scale (11-ASC). Mean and standard deviation are reported in percentages.

**Fig 3:**
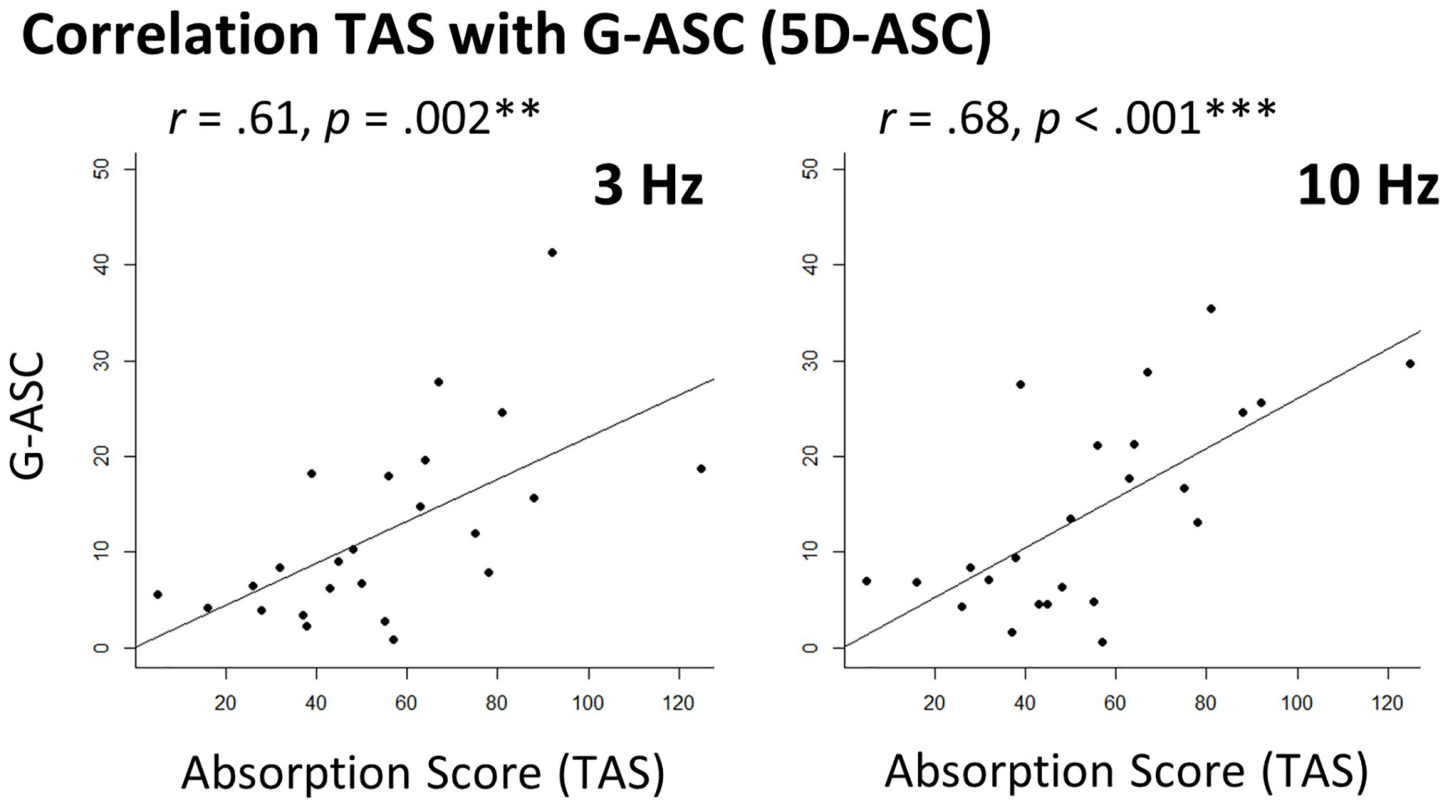
Correlations between personality trait absorption and subjective experiences during Flicker Light Stimulation (FLS). To test the hypothesis that the personality trait of Absorption affects the overall experience of an altered state of consciousness (ASC), we tested for respective correlations in the data. Correlations of participants’ trait of Absorption scores, as assessed with the Tellegen Absorption Scale (TAS) [19], with the G-ASC scores (global mean scores of three 5D-ASC dimensions, reflecting the etiology-independent core of ASCs) are depicted for the 3 Hz and 10 Hz condition. Significant correlations are indicated as *p < .025, **p < .005, and ***p < .0005 (α-threshold Bonferroni-corrected for two comparisons).

Results of the PCI [26] can be found in Fig 3 and Table 2. Explorative analyses did not display significant differences between experimental conditions within any of the assessed dimensions.

**Table 2:** Differences between 3 Hz and 10 Hz conditions in the twelve major and 14 minor dimensions of the PCI [26] as assessed by Wilcoxon signed-rank tests.

PCI Major Dimension	3 Hz		10 Hz		*p*
	*M*	*SD*	*M*	*SD*	
Altered Experience	1.84	1.04	1.89	1.13	0.685
Altered State of Awareness	2.41	1.80	2.71	1.90	0.135
Arousal	1.35	1.73	2.17	1.70	0.056
Attention	4.02	1.08	4.09	1.14	0.603
Imagery	2.68	1.73	3.27	1.57	0.055
Internal Dialogue	2.09	1.90	2.15	2.01	0.733
Memory	4.09	1.67	3.64	1.48	0.298
Negative Affect	0.36	0.70	0.53	0.98	0.890
Positive Affect	1.20	1.16	1.17	0.86	0.836
Rationality	3.57	1.49	3.23	1.48	0.237
Self-Awareness	4.23	1.38	3.97	1.36	0.307
Volitional Control	3.51	1.60	2.94	1.74	0.179
PCI Minor Dimension	3 Hz		10 Hz		*p*
	*M*	*SD*	*M*	*SD*	
Absorption	3.22	1.55	3.67	1.20	0.243
Anger	0.48	1.09	0.39	1.04	0.750
Body Image	1.93	1.73	1.72	1.57	0.375
Direction of Attention	4.55	1.28	4.36	1.33	0.826
Fear	0.17	0.39	0.50	1.17	0.352
Imagery Amount	2.61	2.27	3.48	2.03	0.185
Imagery Vividness	2.76	1.42	3.07	1.38	0.059
Joy	1.52	1.49	1.52	1.51	0.917
Love	1.48	1.50	1.50	1.49	0.800
Meaning	0.67	1.10	0.88	1.25	0.400
Perception	1.86	1.45	2.19	1.72	0.178
Sadness	0.43	0.84	0.70	1.24	0.574
Sexual Excitement	0.48	1.09	0.39	1.04	0.750
Time Sense	3.29	1.76	3.13	1.49	0.572

*Note*. Mean and standard deviation are reported as measured by the inventory (0 minimum, 6 maximum).

Concerning the qualitative analysis of participants’ open reports about their experience during light exposures, S1 Table provides an overview of the number of occurrences of each subjective experience within the experimental conditions. Overall, participants reported that the visual effects of FLS were mainly characterized by the perception of elementary and regular patterns as well as fractal structures: In the 3 Hz condition, ten participants reported the perception of colors and nine the perception of geometric forms or patterns, whereas in the 10 Hz condition 12 participants mentioned color perception and 21 reported having seen forms or patterns during FLS. Also, 13 participants in the 3 Hz and nine participants in the 10 Hz condition mentioned relaxing effects of the FLS.

With regards to time perception, 10 Hz FLS led to a significant overestimation of elapsed time, as indicated by a significant deviation of θ_10 Hz_ (θ = estimated/actual duration; *M* = 1.43 ± *SD* 0.57, n = 4 underestimations) from θ_constant light_ (*M* = 1.20 ± *SD* 0.48, n = 7 underestimations); *p* = .0159, significant after Bonferroni-correction for three comparisons: 0.05/3 = .0166). No significant overestimation was found in the 3 Hz condition, as indicated by θ_3 Hz_ (*M* = 1.28 ± *SD* = 0.50, n = 7 underestimations) when compared to the constant light condition (*p* = .585). Likewise, the difference between the 3 Hz and 10 Hz condition was not significant (*p* = .119). As θ was greater than 1 in all conditions, this indicates that participants overestimated the elapsed time in all conditions. Furthermore, no differences in Time Awareness (*M*_3 Hz_ = 24.52% ± *SD* = 15.00%; *M*_10 Hz_ = 26.28% ± *SD* = 20.93%; rating for “I thought about time”, from 0: ‘*not at all’* to 100: *‘extremely often’*) and Perceived Speed of Time (*M*_3 Hz_ = 48.26% ± *SD* = 24.52%; *M*_10 Hz_ = 49.09% ± *SD* = 23.74%; note that 50% is equivalent to an unaltered perceived time speed), were found between experimental conditions.

PANAS ratings [23] for both positive and negative affect decreased from pre to post FLS (Positive Affect: *M*_3 Hz pre_ = 27.00 ± *SD* = 6.60, *M*_3 Hz post_ = 22.88 ± *SD* = 7.26; *M*_10 Hz pre_ = 26.71 ± *SD* = 6.31, *M*_10 Hz post_ = 24.25 ± *SD* = 5.81; Negative Affect: *M*_3 Hz pre_ = 13.00 ± *SD* = 3.22, *M*_3 Hz post_ = 11.96 ± *SD* = 2.39; *M*_10 Hz pre_ = 12.75 ± *SD* = 3.77, *M*_10 Hz post_ = 12.17 ± *SD* = 3.33), confirmed by a repeated measures ANOVA with a significant main effect of the point of assessment (pre versus post FLS) on participants’ scores on the dimensions Positive Affect (*F*(1,23) = 8.829, *p* = .007) and Negative Affect (*F*(1,23) = 4.865, *p* = .038). No further effects of frequency (Positive Affect: *F*(1,23) = 0.290, *p* = .600; Negative Affect: *F*(1,23) = 0.002, *p* = .968), nor of the interaction of frequency and the point of assessment were found (Positive Affect: *F*(1,23) = 0.841, *p* = .369; Negative Affect: *F*(1,23) = 0.211, *p* = .650). Apart from that, Cohen’s d revealed a medium-sized effect regarding the dimension score of Positive Affect (*d* = 0.59; *95%-CI* = [0.18; 1.01]) and a small effect with respect to Negative Affect (*d* = 0.36; *95%-CI* = [-0.11; 0.83]) in the 3 Hz condition, while effect sizes were small concerning the decrease in Positive Affect (*d* = 0.41; *95%-CI* = [-0.13; 0.94]) and negligible regarding the Negative Affect dimension (*d* = 0.16; *95%-CI* = [-0.17; 0.50]) in the 10 Hz condition.

To test for effects of FLS on divergent thinking, the AUT [24] was applied before and after each FLS session, providing measures for Fluency *(M*_3 Hz pre_ = 7.71 ± *SD* = 2.67, *M*_3 Hz post_ = 7.69 ± *SD* = 2.97; *M*_10 Hz pre_ = 7.40 ± *SD* = 2.00, *M*_10 Hz post_ = 7.17 ± *SD* = 2.54), Flexibility (*M*_3 Hz pre_ = 6.40 ± *SD* = 2.04, *M*_3 Hz post_ = 6.27 ± *SD* = 2.36; *M*_10 Hz pre_ = 5.92 ± *SD* = 1.67, *M*_10 Hz post_ = 5.71 ± *SD* = 1.89) and Originality (*M*_3 Hz pre_ = 9.30 ± *SD* = 1.78, *M*_3 Hz post_ = 9.65 ± *SD* = 1.63; *M*_10 Hz pre_ = 9.57 ± *SD* = 1.70, *M*_10 Hz post_ = 9.25 ± *SD* = 1.93). We performed three 2x2 ANOVAs for each of the AUT scales with the factors point of assessment (pre, post) and condition (3 Hz, 10 Hz). Divergent thinking did not differ between FLS frequencies (no main effect of Fluency: *F*(1,23) = 1.528, *p* = .229; Flexibility: *F*(1,23) = 3.587, *p* = .071; Originality: *F*(1,23) = 0.027, *p* = .870), or time (no main effect of point of assessment: Fluency: *F*(1,23) = 0.130, *p* = .722; Flexibility: *F*(1,23) = 0.329, *p* = .572; Originality: *F*(1,23) = 0.004, *p* = .950). No interaction effects were found either between frequency and the point of assessment (Fluency: *F*(1,23) = 0.146, *p* = .706; Flexibility: *F*(1,23) = 0.024, *p* = .879; Originality: *F*(1,23) = 1.615, *p* = .217).

### Correlations of personality traits and subjective experiences

Investigating the relations between personality factors and the extent of FLS-induced experiences, we found large significant correlations between trait of Absorption and the G-ASC score of the 5D-ASC [25] in both the 3 Hz (*r* (22) = .61, *p* = .002) and 10 Hz (*r* (22) = .68, *p* < .001) conditions. Furthermore, we tested for correlations of the subscales Elementary Imagery and Complex Imagery of the 5D-ASC with participants’ trait of Absorption scores [19]. Complex Imagery did not correlate significantly with Absorption scores (TAS) in both the 3 Hz (*r* (22) = .43, *p* = .034) and the 10 Hz conditions (*r* (22) = .45, *p* = .027) after Bonferroni-correction of the α-threshold. Moreover, we did not find correlations of Elementary Imagery with Absorption scores neither in the 3 Hz (*r* (22) = -.03, *p* = .873), nor in the 10 Hz condition (*r* (22) = .23, *p* = .282). Similarly, we did not find correlations between the personality factor Conscientiousness (NEO-FFI-2) [22] and Elementary Imagery in the 3 Hz (*r* (22) = .32, *p* = .126) or in the 10 Hz condition (*r* (22) = -.24, *p* = .257). Likewise, no correlation between Conscientiousness and Elementary Imagery were found neither in the 3 Hz (*r* (22) = -.05, *p* = .802), nor in the 10 Hz condition (*r* (22) = .20, *p* = .333).

Further correlations between the five personality factors of the NEO-FFI-2 and the G-ASC score (5D-ASC), such as the major dimension Altered State of Awareness and the minor dimension Absorption of the PCI were calculated in an explorative manner. However, none of these correlations were significant after Bonferroni correction for 30 tests (see S2–S4 Tables).

## Discussion

In this study, we systematically assessed the effects of FLS to characterize the phenomenology of flicker-induced ASCs and to allow a comparison to other ASCs phenomena. We compared the effects of two different flicker frequencies by applying well-established inventories which facilitate the comparison of the FLS-induced subjective experiences with those induced by other ASCs induction methods. The effects induced by FLS were dominated by simple visual hallucinations. We did not find significant differences between the effects induced by 3 Hz and 10 Hz FLS. However, we found a general trend for FLS with a frequency of 10 Hz to lead to overall more intense ASCs when compared to 3 Hz FLS. Furthermore, participants’ scores on the personality trait of Absorption positively correlated with the extent of their experienced alterations in consciousness. As hypothesized, distortions of participants’ time perception, as well as certain mood alterations, were among the effects of FLS. We did not find significant effects of FLS on divergent thinking.

### Characterization of the ASCs phenomenology induced by flicker light stimulation

Dimension scores of the 5D-ASC/11-ASC show that the FLS-induced states were particularly characterized by vigilance reduction. This is in line with the results of von Gizycki et al. [13] who reported that FLS was associated with increased sleepiness and a decrease in alertness. Apart from that, 5D-ASC/11-ASC data and participants’ open reports imply that FLS-induced ASCs were mainly associated with perceptual and imaginational alterations, e.g., seeing colors or patterns with closed eyes, being consistent with already existing literature describing an increase of visual imagery [8, 13] and the visual perception of geometric patterns [53] evoked by FLS. In general, 5D-ASC data displayed a trend of higher effects regarding 10 Hz FLS on the G-ASC score, reflecting general consciousness-altering effects. This trend supports the findings of a study by Schwartzman et al. [8] in which participants rated FLS of 10 Hz to be more intense than FLS of 3 Hz. Concerning the dimensions of the PCI, FLS-induced states were also characterized by noticeably high self-awareness as well as focused and mainly self-directed attention.

**Fig 4:**
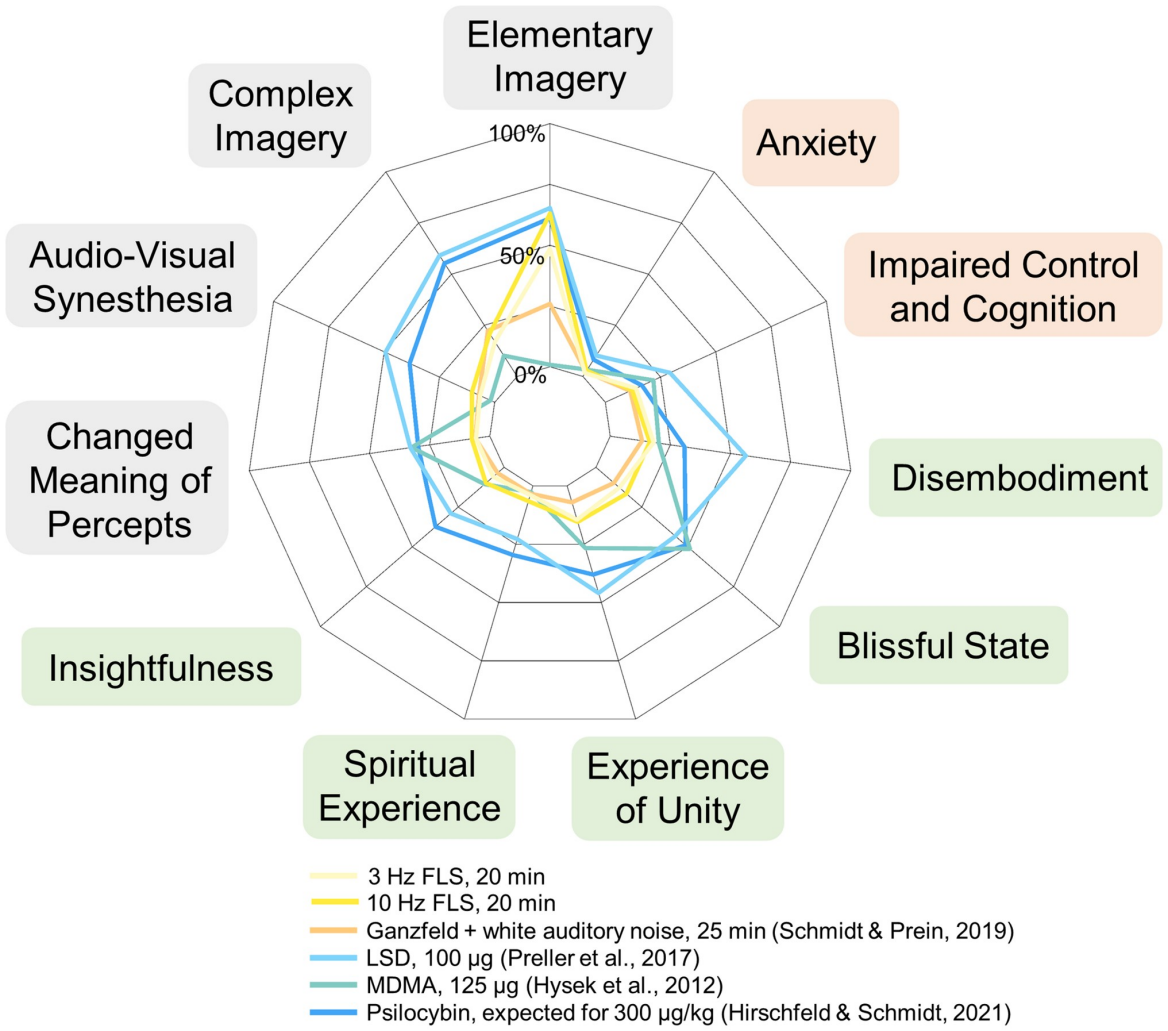
Comparison of ratings of different Altered States of Consciousness (ASCs) experiences induced by pharmacological and non-pharmacological means. All ratings were obtained using the Altered States of Consciousness Rating Scale [25], and results reported according to the 11 factors scheme (11-ASC), constituting subscales of the 5D-ASC dimensions Oceanic Boundlessness (green), Dread of Ego Dissolution (orange), and Visionary Restructuralization (grey). Next to the ratings of 3 Hz and 10 Hz flicker light stimulation from the present study, data from 25 min of exposure to multimodal Ganzfeld stimulation [15] is provided, as well as ratings after the application of 100 μg LSD [57], 125 mg MDMA [59], and the effects of psilocybin at a dose of 300 μg/kg according to a meta-analysis on psilocybin dose-response relationships [5]. Rating scores were taken from the Altered States Database [4].

The application of standardized questionnaires enables comparisons of the subjective ratings of the intensity of FLS-induced effects with those from other studies and even from other ASCs induction methods [4]. Similar subjective effects of different induction methods might point at similar neuronal mechanisms that lead to these experiences. A systematic description and quantification of subjective experiences is a prerequisite for a systematic neuroscientific characterization of underlying neuronal mechanisms. Schwartzman et al. [8] assessed participants’ experiences of 3 Hz and 10 Hz FLS, using items similar to 5D-ASC items, rated on the same visual analog scale as applied in the 5D-ASC. They observed ratings on aspects related to visual effects (“I saw patterns“; “My imagination was extremely vivid“; “Things looked strange“) in a similar range (~75% in the 10 Hz condition; ~50–60% in the 3 Hz condition) as the 5D-ASC ratings observed in the present study. In line with our data, participants gave comparably low ratings for experiences of ego dissolution or mystical-type experiences. To directly compare the obtained 11-ASC ratings for FLS with ratings reported in studies where other methods were applied to induce ASCs, we provide a descriptive comparison in Fig 4. As a comparison with other non-pharmacological methods, we included ratings obtained for multimodal Ganzfeld exposure, comprising visual homogenous light stimulation and auditory stimulation [15, 54]. Comparable ratings were reported for the 5D-ASC/11-ASC dimensions Visionary Restructuralization, indicative of perceptual and imaginational alterations (*M*_*Ganzfeld*_ = 14.03 ± SD = 12.67), as well as Vigilance Reduction, reflecting sleepiness or drowsiness (*M*_*Ganzfeld*_ = 27.12 ± SD = 15.78), and Complex Imagery, indicating the experience of concrete visual impressions of scenes or pictures like e.g., faces (*M*_*Ganzfeld*_ = 22.36 ± SD = 20.09) [15]. However, participants’ ratings observed for FLS-induced experiences in the present study were substantially higher regarding Elementary Imagery, reflecting the perception of more basic visual impressions like colors or patterns (*M*_*Ganzfeld*_ = 25.88 ± SD = 22.92) and lower on the PCI’s factor of Absorption (*M*_*Ganzfeld*_ = 4.23 ± SD = 1.19) than the ratings reported for Ganzfeld-induced experiences [15].

When compared to pharmacologically induced ASCs, FLS-induced experience ratings were comparable to those obtained for low doses of (S)-ketamine on the dimension Vigilance Reduction (*M*_*(S)-ketamine*_ = 28.93 ± SD = 18.3) [55]. With respect to visual effects, ratings of 10 Hz FLS were similarly high as those reported for the consumption of 100 μg LSD regarding Elementary Imagery (*M*_*LSD*_ = 64.01 ± SD = 32.03) [56, 57]. Following a meta-analysis of Hirschfeld and Schmidt [5], the expected effects of 300 μg psilocybin/kg would be of similar magnitude in this dimension (*M*_*psilocybin*_ = 61.1). As the descriptions of the colors, fractal structures, and dynamics of visual effects of FLS share features with descriptions of visual effects induced by dimethyltryptamine (DMT), we also wanted to make a direct comparison with corresponding questionnaire data. As currently no 11-ASC data is available, we could only compare our data with 5D-ASC data. Ratings obtained for DMT fumarate (injections and infusions over 20 min) at a dose of 0.525 mg/kg were slightly higher for Visionary Restructuralization (*M*_*DMT*_ = 35.14 ± SD = 17.85) [58]. Taken together, this comparison suggests that FLS constitutes a non-pharmacological technique whose induced visual effects are rated in a comparable range as effects experienced after the administration of significant doses of psychedelic substances. However, the visual effects appear limited to elementary imagery (i.e., colorful, dynamically changing fractal structures), while complex, hallucination-like visions of meaningful objects were rarely reported in the study at hand (compare also S1 Table).

The effects induced by different techniques that induce alterations in consciousness are well-known to depend not only on the technique but also on the individual and environmental influences [17]. In the context of psychedelic substance use, this is referred to as the effects of ‘set and setting’ [60]. To enable more direct comparisons, future studies could apply within-subjects assessments for different ASCs induction techniques, including pharmacological ones. This could allow identifying whether the experiences induced by a certain technique are predictive of the effects induced by another technique. Such a comparison and prediction could be useful in developing predictors for potential beneficial effects of psychedelics-supported therapeutic approaches [1]. For example, FLS-induced effects might be predictive of a subject’s response to psychedelics, which has been suggested by findings by Brown [61] with respect to LSD. Above that, a similarity of the subjective effects induced by different induction methods might indicate the activation of similar neuronal mechanisms.

Deepening the knowledge with regards to similarities and differences between ASCs induction methods could also inspire future work to explore how specific mechanisms might be therapeutically addressed. Currently, the use of pharmacological methods in the treatment of depression, post-traumatic stress disorder, addiction, and other psychiatric disorders are investigated [62–64]. However, pharmacological methods might come with specific limitations [62, 65]. The potential application of non-pharmacological methods in therapeutic settings thus might also be worth exploring. A standardized comparison between methods constitutes a first step in such an endeavor and serves to motivate the investigation of potential applications of FLS beyond recreational usage.

With regards to pharmacologically and non-pharmacologically induced effects, a combination of FLS with LSD-, psilocybin-, or mescaline-induced ASCs was reported to have led to significantly more reports of color perception in an early study by Hartman and Hollister from 1963 [66]. Equally, the intensity of visually perceived patterns induced by brain stimulations with rectangular electric pulses (1–30 Hz) via electrodes was increased by mescaline, psilocybin, and LSD [67]. However, potentially beneficial effects of combining pharmacological and non-pharmacological ASCs induction methods require closer and careful examination.

In the present study, participants were asked to rate their general experience during FLS. Future work could benefit from a detailed chronologic account considering the variance of experiences, e.g., micro-phenomenological assessment, as recently applied for other ASC experiences [68, 69].

Applying standardized questionnaires further enhances the comparability of the results obtained in different studies. In the study at hand, FLS of 3 and 10 Hz was applied to make our data comparable to a recent report by Schwartzman et al. [8]. Additionally, previous research has indicated that the applied frequency range of FLS has an effect on the diversity and intensity of the induced visual phenomena. Mauro et al. [10] reported most radial illusory percepts at ~10 Hz and spiral percepts to be most pronounced at the slightly higher frequency of ~15Hz. Allefeld et al. [7] assessed various aspects of visual phenomena. These were most strongly pronounced between 10–25 Hz. Reports by Becker and Elliot [70] and Billock and Tsou [71] indicate the strongest illusory percepts in the range of 15 to 30 Hz. With respect to future research, it will be informative to assess the effects of higher flicker frequencies with standardized questionnaire tools, as well.

It must be noted that the study at hand did not include a control condition without light stimulation, as the applied questionnaires ask for changes in experiences that diverge from average everyday experiences. To account for experimental bias, we applied constant light in the first session of the experiment, as previous studies showed that participants tend to give generally higher ratings in first experimental sessions [15]. Future studies could also include a control condition without any light stimulation to control for setting or placebo effects more thoroughly.

### Time perception

On average, participants overestimated the elapsed time in all conditions. However, FLS had an effect on time perception in the 10 Hz condition, manifesting in a significant overestimation of the elapsed time in comparison with the constant light session. This is in line with the suggestion that for time estimations the accumulation of salient perceptual changes is used [72], as FLS of 10 Hz comprised more perceptual changes during exposure than the constant light session and the 3 Hz FLS condition. Jokic et al. [40] observed that the more participants thought about the passage of time in a waiting situation, the higher was their overestimation of the actual duration. In our study, participants were instructed about the time estimation task immediately before light exposure, potentially causing participants to focus on this task. Furthermore, Zakay et al. [73] found a negative correlation between the difficulty of a verbal task and the estimated duration a task took. Participants did not have any other task demanding additional cognitive resources during FLS in the study at hand, which might have led to overestimations. Overall, findings are in accordance with Zakay and Block [74], as well as a meta-analytic review of Block et al. [75], proposing an attentional allocation to the prospective time estimation task to result in higher time estimations. Furthermore, our results are consistent with those of former studies in which participants reported a faster passage of time during multimodal Ganzfeld-induced states [15, 76], which were shown to be characterized by a similar vigilance reduction to the study at hand [15, 54].

### Mood alterations

Anecdotal reports about the use of FLS as a recreational technique indicate that it has a positive effect on mood. To formally test for such effects, we applied the PANAS before and after each flicker session. Participants displayed a significant change (medium-sized decrease) of their Positive Affect in the 3 Hz condition (pre versus post FLS). However, based on a study investigating the effect of FLS on mood states, von Gizycki et al. suggested that levels of affective states remain unaffected, while subjective arousal is reduced by FLS [13]. Moreover, Kumano et al. [77] reported EEG-driven photic stimulation, in which the stimulation frequency was adjusted to the individual alpha frequency, to correlate with improvements of a depressive participant’s mood state and affect. One could hypothesize that a decrease of positive affect might be restricted to lower frequencies, possibly due to the mildly distressing impact of slowly flickering light. In sum, the applied monotonous flicker stimulation had a small negative effect on mood, without major discomfort, making it well-suitable for experimental purposes. Please note that testing for mood alterations was motivated by anecdotal reports of positive effects on mood in recreational settings. Here, we applied monotonous FLS which is different from the highly variable FLS applied in recreational settings.

### Divergent thinking

To test whether FLS had an effect on the performance of divergent thinking, we applied the AUT [24]. We did not find significant effects of FLS on divergent thinking in either of the two experimental conditions. Therefore, no direct mechanistic effects of the rhythmic stimulation were found to contribute to divergent thinking. For testing the existence and nature of potential beneficial effects on divergent thinking, future investigations might use FLS as applied in recreational settings which are thought to have a stronger relaxing impact by immersive, diverse FLS varying in frequency.

### Factors that might influence subjective experiences

To test if personality traits might exhibit an effect on the experiences of FLS, we assessed the trait of Absorption with the TAS [19]. We found large correlations with the G-ASC score (5D-ASC) within both of our experimental conditions. High scores on the trait of Absorption have been linked to highly focused attention “involving a full commitment of available perceptual, motoric, imaginative and ideational resources to a unified representation of the attentional object” [19] which has been hypothesized to facilitate the entrance into ASCs [20]. Our results are in line with those of other studies investigating the role of Absorption in the context of ASCs: Pekala et al. [78] found small correlations of participants’ Absorption with their scores on imaginational and attentional variables, measuring subjective effects of relaxation-meditation. Also, Studerus et al. [17] revealed Absorption as a predictor of alterations in consciousness in response to psilocybin, particularly concerning the dimensions Oceanic Boundlessness and Visionary Restructuralization of the 5D-ASC inventory (small to medium correlations). Thereby our data further highlights the role of the personality trait Absorption, possibly being predictive of how subjective ASC experiences are evaluated.

Within the calculated correlations of the Big Five personality traits (NEO-FFI-2) [22] and the G-ASC score of the 5D-ASC, we neither found a consistent, nor a significant pattern. Similar to Schmidt and Prein [15], no negative correlation was found between Conscientiousness and the evoked amount of imagery, as it had been reported for multimodal Ganzfeld exposure by Wackermann et al. [14] based on a dataset regarding ASCs evoked by the multimodal Ganzfeld method [16].

Recently, the neuronal mechanisms that lead to hallucinations have been discussed in the framework of predictive coding [79]. While normal perceptual processing is thought to rely on the integral interaction of hierarchical processes, a disbalance of bottom-up and top-down signaling could lead to diverse variations in perceptual and higher-order processing [80]. The emergence of hallucinations in pathologic situations is currently thought to result from altered precision and weighting of predictions that are propagated through the cortical hierarchy as top-down signals [81]. Corlett et al. [82] speculate on different mechanisms for simple and complex hallucinations. Simple hallucinations typically comprise the visual impression of colors, flashes, and basic patterns, whereas complex hallucinations are characterized by the perception of concrete phenomena like humans or objects [83]. While the former ones might emerge from altered processing in hierarchically low-level regions, the latter ones are thought to relate to altered top-down influence on higher hierarchical levels. Presumably, FLS will primarily drive a strong bottom-up signal, which could be the reason why the effects are dominated by simple/elementary visuals, potentially emerging from altered processing in low-level visual regions. Complex imagery tends to be reported mainly by participants who also rated high on the personality trait of Absorption. It could be hypothesized that this relates to differences in the higher-order processing (e.g., a broader prior distribution; see also Carhart-Harris and Friston [84]). As our study demonstrates FLS as a suitable tool for experimental research, these potential mechanisms could be tested in future neuroimaging studies. However, it is critical to note that items assessing these dimensions mainly refer to the perception of hallucinatory phenomena with closed eyes or in complete darkness [25], whereas hallucinations associated with clinical disorders like psychosis, dementia, or eye disease are mainly experienced with open eyes [83]. It is thus important to note that some aspects of these phenomena might resemble but they cannot be equated. Also, the free reports provided by our study participants did not indicate that complex hallucinations were among the experiences induced by FLS, as the reported visual phenomena did not reflect complex objects or scenes (see S1 Table). A finer distinction is suggested as the subject of future research.

Future studies on the relationship of set and setting, as well as the role of participants’ expectations, might include additional psychometric or physiological measures to test for additional predictors of subjective experiences, in particular for the nature of hallucinatory perception. As the 5D-ASC was designed to cover a broad range of ASCs phenomena, the assessment of visual effects is limited to the relatively rough distinction between elementary and complex hallucinations. Therefore, future research will strongly benefit from a more detailed assessment of visual effects.

### Outlook

The data presented in the study at hand provide an overview of FLS-induced effects with a focus on ASCs. This detailed characterization increases the comparability of FLS-induced ASCs with the effects induced by other techniques that are used recreationally and for research purposes. On the one hand, our results demonstrate that the visual effects of FLS, in some aspects, are rated to be similarly strong as the effects induced by certain psychedelic substances. On the other hand, the induced spectrum of phenomena is rather limited to visual effects, while other aspects of alterations in consciousness are less robustly induced. However, the application of FLS in recreational settings uses more variability in flicker frequency and light intensity and is often accompanied by music, which together might induce more substantial, relaxing, and personally meaningful states than the applied standardized stimulation in a laboratory setup. Overall, FLS renders a well-suited technique for experimental purposes to investigate the neuronal underpinnings of visually occurring hallucinations in future studies.

## Supporting information

S1 Interview Semi-structured interview on subject screening for studies with Lucia N°03 (English and German versions). (PDF)

S1 Table Results of retrospective participant reports. (PDF)

S2 Table Correlations of personality traits (NEO-FFI-2 and TAS) with G-ASC scores (5D-ASC). (PDF)

S3 Table Correlations of personality traits (NEO-FFI-2 and TAS) with altered state of awareness scores (PCI). (PDF)

S4 Table Correlations of personality traits (NEO-FFI-2 and TAS) with absorption scores (PCI). (PDF)

S1 Data Supplementary study data. (XLSX)
