# Supplementary material for: Altered states phenomena induced by visual flicker light stimulation
Source: PLoS One. 2021 Jul 1;16(7):e0253779. doi: 10.1371/journal.pone.0253779 (PMC8248711; doi:10.1371/journal.pone.0253779)
Supplement: S1 Interview — (PDF) [file pone.0253779.s001.pdf]

Semi-structured interview on participant screening for studies with Lucia N°03

Do you sometimes experience strong fears, compulsions, or panic attacks?

Do you suffer from depression?

Do you have epilepsy?

Have you ever had an idiopathic seizure? If so, has it been examined neurologically?

Do you suffer from migraines?

Do you have difficulties falling or staying asleep?

Have you ever had a psychosis? Have you ever seen or heard things that were not there?

Do you sometimes have the feeling that your perception, your environment is changing?

Is there a first-degree relative in your family (looking back up to two generations) who has schizophrenia?

Have you currently or within the last 3 months been taking psychotropic drugs?

Have you been diagnosed with a neurological or psychiatric illness?

Exclusion criteria:

- History of psychoses
- Current neurological or psychiatric diagnosed diseases
- Currently or up to 3 months ago taking psychotropic drugs
- Acute or chronic lack of sleep

Are you pregnant/can you safely exclude the possibility of pregnancy? (if applicable)

Exclusion criterion:

- Pregnancy

The following questions do not have to be answered in detail. All answers are voluntary. It is sufficient to state whether or not you meet the following criteria.

Have you had any experience with psychedelic substances?

Do you regularly consume alcohol or other substances such as THC, MDMA, etc.?

Please answer with yes or no whether you meet the following criteria:

Exclusion criteria:

- THC up to 1 week before the experiment, before more often than twice a week in the last year
- Psychedelic use in the last two weeks before the study, including microdosing
- Drinking alcohol within the last 12 hours

Halbstrukturiertes Interview zum Proband*innen-Screening für Studien mit Lucia N°03

Erleben Sie manchmal starke Ängste, Zwänge oder Panikattacken?

Leiden Sie an einer Depression?

Leiden Sie an Epilepsie?

Hatten Sie schon einmal einen idiopathischen Krampfanfall? Wenn ja, wurde dieser neurologisch untersucht?

Leiden Sie an Migräne?

Haben Sie Ein- oder Durchschlafstörungen?

Hatten Sie einmal eine Psychose? Haben Sie einmal Dinge gesehen oder gehört, die nicht da waren?

Haben Sie manchmal das Gefühl, dass Ihre Wahrnehmung, Ihre Umwelt sich verändert?

Gibt es in Ihrer Familie (bis zu zwei Generationen rückblickend) eine/n Verwandten ersten Grades, die/der an Schizophrenie erkrankt ist?

Haben Sie aktuell oder in den letzten 3 Monaten Psychopharmaka eingenommen?

Ist bei Ihnen eine neurologische oder psychiatrische Erkrankung diagnostiziert worden?

Ausschlusskriterien:

- Psychosen in der Anamnese
- Aktuelle neurologische oder psychiatrische diagnostizierte Erkrankungen
- Aktuell oder bis vor 3 Monaten Einnahme von Psychopharmaka
- Akuter oder chronischer Schlafmangel

Sind Sie schwanger/können Sie eine Schwangerschaft sicher ausschließen? (falls anwendbar)

Ausschlusskriterium:

- Schwangerschaft

Die Folgenden Fragen müssen nicht detailliert beantwortet werden, Ihre Angaben sind freiwillig. Es ist ausreichend, anzugeben, ob Sie folgende Kriterien erfüllen.

Haben Sie Erfahrungen mit psychedelischen Substanzen gemacht?

Konsumieren Sie regelmäßig Alkohol oder andere Substanzen wie THC, MDMA etc.?

Bitte beantworten Sie mit Ja oder Nein, ob Sie folgende Kriterien erfüllen:

Ausschlusskriterien:

- THC bis zu 1 Woche vor Experiment, davor häufiger als 2x wöchentlich im letzten Jahr
- Psychedelikakonsum in den letzten zwei Wochen vor der Studie, inklusive Microdosing
- Alkoholkonsum innerhalb der letzten 12 Stunden
