# Supplementary material for: Altered states phenomena induced by visual flicker light stimulation
Source: PLoS One. 2021 Jul 1;16(7):e0253779. doi: 10.1371/journal.pone.0253779 (PMC8248711; doi:10.1371/journal.pone.0253779)
Supplement: S1 Table — (PDF) [file pone.0253779.s002.pdf]

Table S1

Content and frequency of each category extracted from retrospective participant reports

Superordinate Category	Extracted Category	Amount Constant Light Session	Amount 3 Hz Session	Amount 10 Hz Session
Visual impressions	Circling patterns		1	1
	Change of light	4	5	2
	Colorful afterimage after the termination of FLS	2		
	Movement of the room/chair	1	2	1
	(Almost) None	3	6	
	Tunnel or spiral	1	2	1
	Colors	10	10	12
	Forms or patterns	5	9	21
	Concrete images	1	1	3
(Forms)	Rasters	1	1	2
	Circles	2	1	3
	Stars	1		1
	Lightning		1	
	Wavelike patterns	1		
	Light rings		1	
	Light points			1
	Heart	1		
(Colors)	Red	1	9	4
	Green	1	1	2
	Blue	1	3	5
	Yellow	4	4	
	Orange	3	1	3
	Beige	1		
	Purple		1	
	Black		2	3
	Grey			1
	White			2
Thoughts	Thoughts about everyday life	8	6	
	Very few thoughts	6	6	4
	(Day-)dreaming	3	1	2
	Re-experiencing situations	6		
	Thoughts about problems	1		
	Finding new ideas and solutions	1		1
	Not able to remember specific thoughts	1	1	2

Table S1

Continued

Superordinate Category	Extracted Category	Amount Constant Light Session	Amount 3 Hz Session	Amount 10 Hz Session
Thoughts (continued)	Interrupted thoughts	1		1
	Problems were less frightening	1		
	Erotic thoughts		1	
	Thinking up stories		2	
	Thinking of certain places or persons	2		1
	Wandering thoughts	1	2	2
	Clearer thoughts	1		
	Child´s emotion	1		
	Seeing thoughts as pictures		1	
	Excessive stream of thoughts		2	
Arousal and emotions	Dizziness		3	1
	Nausea		1	
	Restlessness		2	2
	Tiredness	11	4	4
	Wakefulness	3	1	1
	(Feeling like) falling asleep	12	5	4
	Relaxation	12	13	9
	Compulsion to move	1	1	
	Unpleasant Feeling	1	4	9
	Feeling safe	4		
	Feeling concentrated after the termination of FLS	1		
	Feeling spaced out after the termination of FLS	1		1
	Habituation to the (flicker) light during session	6	9	12
	Trancelike/meditative state	3	1	1
	Liberating feeling	1		1
	Sadness	1		
Bodily perceptions	Tingling	1	1	
	Limbs felt paralyzed			1
	Convulsions		1	1
	Twitch of the eyes	2	2	3
	Watery eyes		1	1
	Itchy eyes			1
	Decrease in bodily perception	1		
	Bodily awareness	1		
	Body felt heavy		2	1
	Feeling of being able to leave body		1	
	Feeling of floating	1		

Table S1

Continued

Superordinate Category	Extracted Category	Amount Constant Light Session	Amount 3 Hz Session	Amount 10 Hz Session
Perception of temperature	Warmth	10	1	
	Coldness to the end of the session	1	1	
Time and space perception	Altered time perception	1	3	1
	Altered space perception		2	
Associations	Sunlight	7	2	
	Rollercoaster			2
	Outer space		1	
	Moving spotlight		1	
Other	Better perception of sound	1		

Note. For each session, the number of participants whose reported experiences were classified into the extracted categories is given. Categories are divided into superordinate categories representing the aspect affected by the experience. The superordinate category 'Visual alterations' is further divided into more specific visual perceptions.
