# Supplementary material for: Altered states phenomena induced by visual flicker light stimulation
Source: PLoS One. 2021 Jul 1;16(7):e0253779. doi: 10.1371/journal.pone.0253779 (PMC8248711; doi:10.1371/journal.pone.0253779)
Supplement: S2 Table — (PDF) [file pone.0253779.s003.pdf]

Table S2

Correlations of personality traits (NEO-FFI-2 and TAS) with G-ASC scores (5D-ASC)

Personality Trait	3 Hz				10 Hz			
	Correlation with G-ASC	*t*	*df*	*p*	Correlation with G-ASC	*t*	*df*	*p*
Absorption	0.61	3.59	22	.002	0.68	4.32	22	.001
Neuroticism	0.18	0.87	22	.395	0.15	0.70	22	.490
Extraversion	-0.09	-0.43	22	.674	0.21	0.99	22	.335
Openness to Experience	0.22	1.06	22	.300	0.47	2.51	22	.020
Agreeableness	-0.02	-0.11	22	.915	0.10	0.46	22	.651
Conscientiousness	-0.04	-0.20	22	.843	-0.06	-0.29	22	.776

Note. Pearson product-moment correlations were calculated between the global G-ASC score of the Altered States of Consciousness (ASC) Rating Scale (5D-ASC; Dittrich, Lamparter, & Maurer, 2006) in the 3 Hz FLS condition and the 10 Hz FLS condition with the personality trait Absorption, as assessed with the Tellegen Absorption Scale (TAS; Tellegen & Atkinson, 1974), and the Big Five personality traits, as assessed with the NEO-FFI-2 (Costa & McCrae, 1989).
