# Supplementary material for: Altered states phenomena induced by visual flicker light stimulation
Source: PLoS One. 2021 Jul 1;16(7):e0253779. doi: 10.1371/journal.pone.0253779 (PMC8248711; doi:10.1371/journal.pone.0253779)
Supplement: S3 Table — (PDF) [file pone.0253779.s004.pdf]

Table S3

Correlations of personality traits (NEO-FFI-2 and TAS) with Altered State of Awareness scores (PCI)

Personality Trait	3 Hz				10 Hz			
	Correlation with Altered State of Awareness	*t*	*df*	*p*	Correlation with Altered State of Awareness	*t*	*df*	*p*
Absorption	0.32	1.60	22	.123	0.39	1.96	22	.062
Neuroticism	-0.09	-0.43	22	.670	0.03	0.14	22	.891
Extraversion	0.32	1.58	22	.129	0.13	0.62	22	.540
Openness to Experience	0.05	0.22	22	.828	0.10	0.46	22	.649
Agreeableness	-0.12	-0.58	22	.570	0.06	0.30	22	.768
Conscientiousness	-0.04	-0.17	22	.864	-0.18	-0.86	22	.398

Note. Pearson product-moment correlations were calculated for the major dimension Altered States of Awareness of the Phenomenology of Consciousness Inventory (PCI; Pekala, 1991) in the 3 Hz FLS condition and the 10 Hz FLS condition with the personality traits Absorption, as assessed with the Tellegen Absorption Scale (TAS; Tellegen & Atkinson, 1974), and the Big Five personality traits, as assessed with the NEO-FFI-2 (Costa & McCrae, 1989).
