# Supplementary material for: Altered states phenomena induced by visual flicker light stimulation
Source: PLoS One. 2021 Jul 1;16(7):e0253779. doi: 10.1371/journal.pone.0253779 (PMC8248711; doi:10.1371/journal.pone.0253779)
Supplement: S4 Table — (PDF) [file pone.0253779.s005.pdf]

Table S4

Correlations of personality traits (NEO-FFI-2 and TAS) with Absorption scores (PCI)

Personality Trait	3 Hz				10 Hz			
	Correlation with Absorption	*t*	*df*	*p*	Correlation with Absorption	*t*	*df*	*p*
Absorption	0.34	1.69	22	.105	0.44	2.28	22	.033
Neuroticism	0.36	1.80	22	.086	0.00	0.00	22	1
Extraversion	-0.26	-1.26	22	.220	0.18	0.85	22	.404
Openness to Experience	0.33	1.63	22	.117	0.19	0.93	22	.363
Agreeableness	-0.04	-0.18	22	.858	0.27	1.32	22	.200
Conscientiousness	-0.24	-1.15	22	.261	0.02	0.11	22	.915

Note. Pearson product-moment correlations were calculated for the minor dimension Absorption of the Phenomenology of Consciousness Inventory (PCI; Pekala, 1991) in the 3 Hz FLS condition and the 10 Hz FLS condition with the personality traits Absorption, as assessed with the Tellegen Absorption Scale (TAS; Tellegen & Atkinson, 1974), and the Big Five personality traits, as assessed with the NEO-FFI-2 (Costa & McCrae, 1989).
